# Decreased mitochondrial metabolic requirements in fasting animals carry an oxidative cost

**DOI:** 10.1111/1365-2435.13125

**Published:** 2018-05-29

**Authors:** Karine Salin, Eugenia M. Villasevil, Graeme J. Anderson, Sonya K. Auer, Colin Selman, Richard C. Hartley, William Mullen, Christos Chinopoulos, Neil B. Metcalfe

**Affiliations:** ^1^ Institute of Biodiversity, Animal Health and Comparative Medicine University of Glasgow Glasgow UK; ^2^ School of Chemistry University of Glasgow Glasgow UK; ^3^ Institute of Cardiovascular and Medical Sciences University of Glasgow Glasgow UK; ^4^ Department of Medical Biochemistry Semmelweis University Budapest Hungary; ^5^ MTA‐SE Lendület Neurobiochemistry Research Group Budapest Hungary

**Keywords:** high‐resolution respirometry, in vivo, liver atrophy, MitoB probe, mitochondrial respiratory state

## Abstract

1. Many animals experience periods of food shortage in their natural environment. It has been hypothesised that the metabolic responses of animals to naturally‐occurring periods of food deprivation may have long‐term negative impacts on their subsequent life‐history.
2. In particular, reductions in energy requirements in response to fasting may help preserve limited resources but potentially come at a cost of increased oxidative stress. However, little is known about this trade‐off since studies of energy metabolism are generally conducted separately from those of oxidative stress.
3. Using a novel approach that combines measurements of mitochondrial function with in vivo levels of hydrogen peroxide (H_2_O_2_) in brown trout (*Salmo trutta*), we show here that fasting induces energy savings in a highly metabolically active organ (the liver) but at the cost of a significant increase in H_2_O_2_, an important form of reactive oxygen species (ROS).
4. After a 2‐week period of fasting, brown trout reduced their whole‐liver mitochondrial respiratory capacities (state 3, state 4 and cytochrome *c* oxidase activity), mainly due to reductions in liver size (and hence the total mitochondrial content). This was compensated for at the level of the mitochondrion, with an increase in state 3 respiration combined with a decrease in state 4 respiration, suggesting a selective increase in the capacity to produce ATP without a concomitant increase in energy dissipated through proton leakage**.** However, the reduction in total hepatic metabolic capacity in fasted fish was associated with an almost two‐fold increase in in vivo mitochondrial H_2_O_2_ levels (as measured by the MitoB probe).
5. The resulting increase in mitochondrial ROS, and hence potential risk of oxidative damage, provides mechanistic insight into the trade‐off between the short‐term energetic benefits of reducing metabolism in response to fasting and the potential long‐term costs to subsequent life‐history traits.

## Foreign Language Abstract Résumé

1. Les restrictions alimentaires sont courantes dans le milieu naturel et peuvent impacter de nombreux animaux. Il a été émis l'hypothèse que les animaux, face à ces épisodes de restriction alimentaire, mettaient en place des réponses métaboliques pouvant affecter leurs histoires de vie future.
2. En particulier, si une diminution des besoins énergétiques lors du jeûne peut contribuer à préserver les réserves de l'animal cela peut néanmoins entraîner une augmentation du stress oxydant. Ce type de compromis n'a toutefois pas encore été démontré car l'étude du métabolisme énergétique est généralement réalisée séparément de celle du stress oxydant.
3. Par une nouvelle approche combinant des mesures du fonctionnement mitochondrial et des niveaux in vivo de peroxyde d'hydrogène (H2O2) chez la truite commune (*Salmo trutta*), nous montrons ici que le jeûne entraîne une économie d'énergie dans un tissu métaboliquement très actif tel que le foie, mais au coût d'une augmentation significative en H2O2, une forme majeure des espèces réactives de l'oxygène.
4. Après deux semaines de jeûne, les truites communes ont réduit leurs capacités respiratoires mitochondriales (état 3, état 4 et l'activité de la cytochrome c oxydase) principalement du fait d'une réduction de la taille du foie (et donc du nombre total de mitochondries). Une compensation a été observée au niveau de la mitochondrie. Cela se traduit par une augmentation de la respiration en état 3 et une diminution concomitante de celle en état 4, suggérant une augmentation sélective des capacités de production de l'ATP sans augmentation parallèle de l'énergie dissipée par la fuite de protons. La diminution des capacités métaboliques du foie chez les poissons à jeun était associée in vivo à des niveaux quasiment doubles de H2O2 mitochondriaux (mesurés par la sonde MitoB).
5. Cette augmentation en espèces réactives de l'oxygène dans les mitochondries, avec son risque inhérent de dommages oxydatifs, apporte une vision mécanistique du compromis entre les bénéfices énergétiques à court terme d'une réduction métabolique en réponse au jeûne et les possibles coûts à long terme sur leurs traits histoires de vie futurs.

A http://onlinelibrary.wiley.com/doi/10.1111/1365-2435.13125/suppinfo is available for this article.

## 1 INTRODUCTION

Many animals live in environments where food abundance varies over time. The challenge during episodes of low food availability is to maintain physiological function while relying primarily on internal energy stores (Wang, Hung, & Randall, 2006). To meet that challenge, animals utilize physiological responses that reduce their metabolic requirements and thus enhance their chance of survival (Secor & Carey, 2016). A central component of such responses is the alteration of mitochondrial energy metabolism (Bermejo‐Nogales, Calduch‐Giner, & Pérez‐Sánchez, 2015; Chausse, Vieira‐Lara, Sanchez, Medeiros, & Kowaltowski, 2015; Monternier, Marmillot, Rouanet, & Roussel, 2014). However, mitochondria are also a major source of reactive oxygen species (ROS) that have the potential to cause oxidative damage (Brand, 2016). Temporary reductions in mitochondrial energy requirements, while providing short‐term energetic benefits, could potentially lead to associated increases in ROS levels, resulting in the long‐term costs of oxidative stress (Schull et al., 2016; Sorensen et al., 2006), potentially faster organismal senescence and hence constraints on future life history (Dowling & Simmons, 2009; Midwood, Larsen, Aarestrup, & Cooke, 2016; Monaghan, Metcalfe, & Torres, 2009; Selman, Blount, Nussey, & Speakman, 2012; Speakman et al., 2015). However, surprisingly little is known about these interactions as studies of mitochondrial energetics are generally conducted separately from those of ROS production (Sorensen et al., 2006; Zhang, Wu, & Klaassen, 2013; but see Brown & Staples, 2011; Chausse et al., 2015).

One of the main functions of the mitochondria is to produce ATP through oxidative phosphorylation. This process involves the pumping of protons by the electron transport chain (ETC) from the matrix to the intermembrane space of the mitochondria, a process that consumes oxygen. The accumulation of protons within the intermembrane space generates an electrical (ΔΨ) and chemical (ΔpH) gradient across the inner mitochondrial membrane (IMM). The gradient causes protons to flow back across the IMM to the matrix through the ATP synthase complex, driving the production of ATP; this process is estimated in vitro as the state 3 respiration rate (Chance & Williams, 1955). However, dissipation of the proton gradient occurs not only during ATP production but also as a result of the leakage of protons directly across the IMM (Brand & Nicholls, 2011; Chance & Williams, 1955). This leakage must be continually offset by the activity of the ETC. complexes, a compensatory process (estimated in vitro as the state 4 respiration rate) which consumes a significant amount of both oxygen and substrate: for example, the futile cycle of proton pumping and proton leakage within liver mitochondria is estimated to account for *c*. 20% of whole‐animal oxygen consumption in rats (Rolfe & Brand, 1997).

Fasting can change rates of both state 3 and state 4 respiration (Bobyleva‐Guarriero, Hughes, Ronchetti‐Pasquali, & Lardy, 1984; Brown & Staples, 2010; Chausse et al., 2015; Guderley, Lapointe, Bédard, & Dutil, 2003; Sorensen et al., 2006), which in turn is likely to provide energetic benefits to an organism, but this may come at a cost in terms of oxidative stress (Geiger, Kauffmann, Le Maho, Robin, & Criscuolo, 2012; Pascual, Pedrajas, Toribio, López‐Barea, & Peinado, 2003; Sorensen et al., 2006). One of the key parameters determining the generation of mitochondrial ROS is ΔΨ, with a higher gradient leading to greater production of ROS (Korshunov, Skulachev, & Starkov, 1997; Miwa & Brand, 2003). As a higher ΔΨ also potentially increases the efficiency of cellular energy transduction (Harper, Dickinson, & Brand, 2001), we predicted that food shortage may prompt an increase in ΔΨ but with a consequent increase in mitochondrial ROS production, and hence greater potential for oxidative damage. Until recently, it has proved technically impossible to measure ROS levels in living multicellular animals, so that ROS levels in relation to nutritional state has instead been evaluated through in vitro assays (Sanz, 2016). These studies have been inconclusive, reporting positive (Sharma, Agrawal, & Roy, 2011; Sorensen et al., 2006; Zhang et al., 2013), negative (Brown & Staples, 2011) or no (Chausse et al., 2015) effect of fasting on ROS production, possibly reflecting the fact that in vitro ROS assays are unreliable estimates of pro‐oxidant levels in living animals (Barja, 2007; Goncalves, Quinlan, Perevoshchikova, Hey‐Mogensen, & Brand, 2014; Sanz, 2016).

The present experiment is the first to integrate measurements of mitochondrial metabolic demand with in vivo ROS levels to examine whether *reductions* in metabolism in response to food shortage lead to *increases* in oxidative stress. We determined mitochondrial respiratory capacity alongside mitochondrial membrane potential and levels of hydrogen peroxide (H_2_O_2_, a major form of ROS) in brown trout (*Salmo trutta*). We investigated this trade‐off in the liver, the organ that displays the most rapid and dramatic changes during fasting (Guderley et al., 2003; Wang et al., 2006). Specifically, we tested whether plasticity in mitochondrial respiratory capacities (state 3 and state 4) and density (estimated from cytochrome *c* oxidase (COX) activity) in response to food shortage causes a reduction in the liver's requirements for oxygen, and in turn energy substrates. Mass‐specific, COX‐normalized (to correct for variation in mitochondrial density, as in Salin, Auer, Anderson, Selman, and Metcalfe (2016); Salin, Auer, Rudolf, et al. (2016)) and whole‐tissue approaches were employed to determine the effects of fasting on mitochondrial oxidative capacities at different levels of biological organization. Moreover, we tested whether the mitochondrial changes that occur during a period of fasting are associated with increased ROS levels estimated using the recently developed MitoB probe that measures the level of mitochondrial H_2_O_2_ in living organisms (Cochemé et al., 2011; Salin et al., 2015, 2017). Our findings demonstrate that brown trout experiencing a simulated natural period of food shortage show dramatic reductions in liver size and hence liver aerobic metabolism. However, these changes are associated with significantly increased hepatic mitochondrial H_2_O_2_ levels and hence potentially the risk of increased oxidative stress.

## 2 MATERIALS AND METHODS

### 2.1 Experimental animals

Brown trout fry were obtained from a commercial hatchery (Howietoun, UK) in summer 2015 and moved to a freshwater recirculation system at the University of Glasgow. Here, the fish were maintained under an 8‐hr light: 16‐hr dark photoperiod at 12C and fed daily in excess with trout pellets (EWOS, West Lothian, UK). In January 2016, twenty‐four fish were transferred to individual compartments within a stream tank system that allowed us to control the food intake of individual fish while maintaining them in identical conditions of water temperature and quality. Fish were moved to this system in batches of two fish per day as final measurements of mitochondrial properties could only be conducted on two fish per day; therefore, all fish were exposed to the diet treatments for the same length of time. The fish were acclimated in the stream system for a week and fed daily to excess prior to the start of the experiment. Half of the fish were then randomly allocated to the same ad libitum ration as they had previously experienced, while the other half were deprived of food (*N* = 12 fish per group). Fish were held on these treatments for 2 weeks, which is a realistic period of food shortage that might be encountered by brown trout in the wild (Bayir et al., 2011; Huusko et al., 2007). All individuals were measured for body mass (± 1 mg) at the start and the end of the 2‐week food treatment.

### 2.2 Measurement of hydrogen peroxide levels

Hydrogen peroxide (H_2_O_2_) levels were measured in vivo using the MitoB probe. This probe is injected into the animal and becomes concentrated in the mitochondria where it is converted to an alternate stable form, MitoP, in the presence of H_2_O_2_. As such, the ratio of MitoP to MitoB is proportional to mitochondrial H_2_O_2_ levels (Cochemé et al., 2012; Salin et al., 2015, 2017). On day 12 of the food treatment, each fish was briefly anaesthetised (50 mg/ml benzocaine diluted in water) and given an intraperitoneal injection of a standard dose of MitoB solution (100 μl of 504 μM MitoB, i.e. 50 nmol/fish), previously diluted in 0.7% (v/v) ethanol and sterile saline solution 0.9% (w/v) NaCl/H_2_O. As the size of the fish ranged from 9.4 to 26.0 g (measured at sacrifice), the initial MitoB concentration varied up to threefold among fish, but previous studies have shown that this range of initial MitoB concentrations does not affect the subsequent measure of H_2_O_2_ levels (Salin et al., 2015, 2017). The injected fish were then returned to their tanks and culled 48 hr later (48.1 ± 0.1 hr; time of day of culling: 09:30 ± 00:01 hr) after having been deprived of food overnight. Their livers were immediately dissected, weighed (0.001 g precision, Explorer^®^ balance) and divided into three aliquots that were also weighed. Two aliquots were then transferred to 1 ml of ice‐cold respirometry buffer for subsequent measurement of mitochondrial properties (see below). The third aliquot was immediately flash‐frozen in liquid nitrogen and stored at −70°C for subsequent extraction and quantification of MitoB and MitoP (Salin et al., 2015, 2017). After extraction of the Mito compounds, the relative levels of MitoP and MitoB were determined by high‐performance liquid chromatography‐tandem mass spectrometer, allowing estimation of average mitochondrial H_2_O_2_ levels over the 48‐hr period from the ratio of MitoP to MitoB (Salin et al., 2015, 2017).

### 2.3 Mitochondrial homogenate preparation

A liver aliquot from each fish (mean ± *SE* across all treatments: 43.08 ± 2.02 mg) preserved in respirometry buffer (0.1 mM EGTA, 15 μM EDTA, 1 mM MgCl_2_, 20 mM Taurine, 10 mM KH_2_PO_4_, 20 mM HEPES, 110 mM D‐sucrose, 60 mM lactobionic acid, 1 g/L bovin serum albumin essentially free fatty acid, pH 7.2 with KOH) was shredded using microdissecting scissors, and the shredded solution then homogenized with a Potter‐Elvehjem homogenizer (three passages). Validations of the methods are described in (Salin, Auer, Anderson, et al., 2016; Salin, Auer, Rudolf, et al., 2016). The homogenate was then diluted further in respirometry buffer to obtain the desired final concentration (mean ± *SE*: 5.06 ± 0.03 mg/ml). The entire procedure was carried out on ice and completed within 30 min of the fish being culled.

### 2.4 High‐resolution mitochondrial respiration rate

Rates of oxygen consumption (*J*O_2_ in pmol O_2_/s) were measured using an Oxygraph 2‐k high‐resolution respirometer equipped with two measurement chambers and then analysed using datlab software (Oroboros Instruments, Innsbruck Austria). The oxygen electrodes were first calibrated at two points: air‐saturated buffer and zero oxygen. The air saturation calibration was achieved by adding respiratory buffer and then allowing oxygen concentration to stabilize, while the zero oxygen calibration was achieved by adding saturating dithionite. Oxygen flux was corrected for instrumental background oxygen flux (Pesta & Gnaiger, 2012). Part of the liver homogenate from each fish was added to one of the two measurement chambers of an oxygraph immediately following preparation; both fish from each processing pair were measured in parallel. The remaining part of the liver homogenate was preserved on ice for use in a replicate trial of measurement of mitochondrial respiration. After addition of homogenate to the respiration chamber at 12°C, pure oxygen gas was added to reach a concentration of 550 μM. Magnesium green (2.1 μM) was present in the respirometry chambers following the design of another project (Salin, Villasevil, et al., 2016)).

The titration protocol was as follows: first, the tricarboxylic acid cycle was reconstituted by adding pyruvate (5 mM) and malate (0.5 mM) to support electron entry to complex I, and succinate (10 mM) to support electron entry to complex II. State 3 was reached by adding a saturating concentration of ADP (2 mM ADP). State 4 was then induced by adding carboxyatractyloside (4 μM), an inhibitor of adenine nucleotide translocator. The opening of the permeability transition port by the carboxyatractyloside was prevented by the absence of free calcium in the buffer (Bernardi, Rasola, Forte, & Lippe, 2015). Addition of complex I inhibitor (0.5 μM rotenone) and complex III inhibitor (2.5 μM antimycin A) determined residual oxygen consumption (ROX), which was then subtracted from all other values. Finally, COX was measured by adding ascorbate (8 mM) and N,N,N',N'‐tetramethyl‐p‐phenylenediamine dihydrochloride (TMPD, 0.5 mM). Cytochrome *c* oxidase, an IMM enzyme involved in the ETC, is a marker of mitochondrial content and is highly correlated with mitochondrial respiratory capacity (Larsen et al., 2012). The auto‐oxidation of TMPD can generate a “chemical background” consumption of oxygen which is not due to the biological sample, so the measured mass‐specific COX activities were affected by a noise—but one that was constant across all samples. The chemical background can normally be quantified by measuring the oxygen flux after inhibition of COX with cyanide, but in this study, this was not feasible because the use of pyruvate substrate reverses the inhibition by cyanide.

The second trial was identical to the first one but started 2 hr later, using the remaining liver homogenate and the other measurement chamber (to control for any interchamber difference in readings). No effect of the choice of measurement chamber on mass‐specific *J*O_2_ was found. We expressed mass‐specific state 3 and state 4 *J*O_2_ values and COX activity as pmoles of O_2_ s^−1^ mg^−1^ wet weight of liver for each replicate. Measurements of the oxidative capacities were reproducible (state 3: ICC *r *=* *0.836, *df* = 23, *p *<* *0.001; state 4: ICC *r *=* *0.542, *df* = 23, *p *=* *0.008; COX activity: ICC *r *=* *0.771, *df* = 21, *p *=* *0.008). The mean mass‐specific state 3 and state 4 *J*O_2_ values and COX activity were determined for each fish by averaging the values from the replicates.

### 2.5 Measurement of mitochondrial membrane potential (ΔΨ)

A safranin probe was used to infer the mitochondrial membrane potential (Åkerman & Wikström, 1976). The safranin fluorescent signal was first calibrated in an independent experiment with valinomycin and by stepwise addition of KCl (Krumschnabel, Eigentler, Fasching, & Gnaiger, 2014). The protocol for measuring Δψ was performed at 12°C, simultaneously for the two fish in each processing pair. The oxygraph was equipped with a fluorescence module with two sensors with filter set for safranin (excitation at 495 mm and emission detection at 587 nm). The second aliquot of liver tissue (mean ± *SE*: 34.75 ± 1.88 mg), previously kept on ice, was homogenized as described above just before the Δψ assay. Homogenate (5 mg/ml) was added to the chamber of the oxygraph 2‐k respirometer together with pure oxygen gas (to reach a concentration of 550 μM). The mitochondrial membrane potential was measured in the presence of safranin (3.75 μM) under different mitochondrial states (state 3 and state 4) using the same titration as in the “high‐resolution mitochondrial respiration rate” section above. In our experimental conditions, as reported in a previous study (Krumschnabel et al., 2014), inhibitory effects of safranin on mitochondrial oxygen consumption were obvious even at a very low concentration of safranin (1 μM). For this reason, mitochondrial respiratory capacities were determined in a safranin‐free environment, but the rates of oxygen consumption in the presence of safranin were used to validate the response and stabilization of mitochondrial activity.

### 2.6 Statistical analysis

Paired *t* tests were used to test for changes in body mass of the fish between the start and the end of the 2‐week treatment period. Linear mixed models (LMM) were used to test whether fed and fasted fish differed significantly in their body and liver masses. All models included pair as a random effect to account for the order in which fish entered the experiment. The analysis of liver mass also included body mass as a covariate. This approach was used instead of calculating the hepatosomatic index (HSI: (liver mass/body mass)*100) as the mass of the liver was not isometrically related to body mass, but we refer to the HSI in the results section as a means of comparing differences in liver mass after correction for body mass.

We then used LMMs to examine mitochondrial oxidative capacities in three different methods of calculation that reveal different aspects of the responses to food shortage. First, we tested whether mean mass‐specific state 3 and state 4 *J*O_2_ values and COX activity (pmol O_2_ s^−1^ mg^−1^ liver) differed between food treatment groups. Second, we tested whether treatment differences in mass‐specific *J*O_2_ were independent of variation in mitochondrial content. The density of mitochondria in many organs is plastic and can change substantially as a result of fasting (Frick, Bystriansky, Ip, Chew, & Ballantyne, 2008), so we corrected for its potential effects on both mass‐specific state 3 and state 4 respiratory capacities by including COX activity as a covariate in the above models (Salin, Auer, Anderson, et al., 2016; Salin, Auer, Rudolf, et al., 2016). Slopes of mass‐specific *J*O_2_ as a function of mass‐specific COX were homogenous among treatment groups in this second model. Third, we tested for food treatment differences in respiration rates scaled up to the whole‐liver level (pmol O_2_ s^−1^ mg^−1^ liver × liver size in mg) as the size of organs relative to body size can also change substantially following fasting (Secor & Carey, 2016).

Finally, we analysed the effect of food treatment on membrane potential and MitoP/MitoB ratio using LMM approaches. The models included the membrane potential or MitoP/MitoB ratio as the dependent variable and food treatment as factorial predictor. Analyses are based on a sample size of 10‐12 fish per group and were performed in IBM SPSS Statistics 21 (SPSS Inc., Chicago, IL, USA); the level of significance was set to *p *<* *0.05, and all means are presented ± *SE*.

## 3 RESULTS

Fasted and fed groups did not differ with respect to body mass at the beginning of the experiment (12.14 ± 0.61 vs. 12.00 ± 0.57 g in the fasted and fed groups respectively, *F*_1,11_ = 0.062, *p *=* *0.81). Food deprivation led to an average 11% reduction in body mass after 2 weeks (final mass of fasted group: 10.85 ± 2.14 g; paired *t* test: *t *=* *11.94, *df* = 11, *p *<* *0.001). Over the same period the fed trout gained mass (final mass fed group: 16.50 ± 3.80 g; paired *t* test: *t *=* *7.00, *df* = 11, *p *<* *0.001). As a result, the body mass of fasted and fed fish significantly differed at the end of the experimental procedure (*F*_1,11_ = 36.99, *p *<* *0.001). These changes in body mass were reflected in the liver mass of the two groups: at the end of the treatment, the liver of the fasted fish was one‐third the size of that of fed fish (93.56 ± 6.33 vs. 303.33 ± 27.73 mg in the fasted and fed groups, respectively; *F*_1,11_ = 64.40, *p *<* *0.001), a difference that remained highly significant after correcting for body mass (*F*_1,20_ = 47.26, *p *<* *0.001). As a result, the HSI of fasted trout was less than half that of fed trout of the same body size (HSI (%) = 0.86 ± 0.03 vs. 1.83 ± 0.11 for the fasted and fed group, respectively).

**Figure 1:**
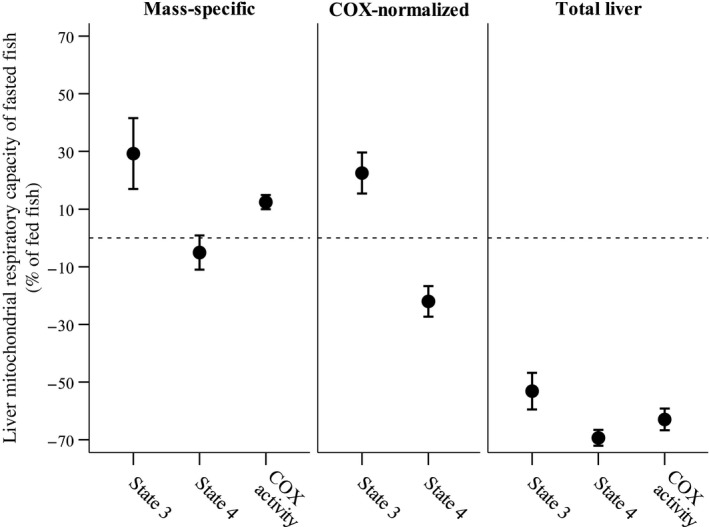
Effect of a 2‐week fasting period on liver mitochondrial respiratory capacities in brown trout. Values for fasted fish are expressed as a percentage of those measured in continuously fed fish and are plotted separately for method of calculation per unit mass (mass‐specific) after accounting for cytochrome *c* oxidase activity (COX‐normalized) and scaled up to the whole‐liver level (total liver). See Table 1 for statistical analysis

Mass‐specific state 3 *J*O_2_ and mass‐specific COX activity were significantly higher in the mitochondria of fasted compared to fed fish, while mass‐specific state 4 *J*O_2_ was slightly—but not significantly lower (Table 1, Figure 1). After accounting for mitochondrial density (i.e. COX activity; Supporting Information Table S1), state 3 *J*O_2_ in the mitochondria of fasted trout was significantly higher than that of fed trout, whereas the corresponding state 4 *J*O_2_ was significantly lower (Table 1, Figure 1). However, the effects of the diet treatment appeared very different when mitochondrial respiratory capacities were expressed in terms of the entire liver: both total liver state 3 and state 4 respiratory capacities were significantly reduced in fasted trout (Table 1, Figure 1). Total liver COX activity was also significantly lower in fasted relative to fed trout (Table 1, Figure 1), suggesting a reduction in the total mitochondrial content of the liver.

**Table 1:** Results from linear mixed models comparing mitochondrial parameters of the livers of brown trout (*Salmo trutta*) that were either fasted or fed over a 2‐week period. Cytochrome *c* oxidase (COX) refers to cytochrome *c* oxidase. COX‐normalized refers to models that control for effects of COX activity: partial residuals of state 3 and state 4 respiration rates were calculated as COX‐normalized values, so that the reported respiration rates reflect the values predicted for each individual fish as if its COX activity was equal to the mean COX activity across all treatments (33.50 pmol O_2_ s^−1^ mg^−1^ liver). Means are presented ± *SE*. *p* Values for significant effects are in bold. *N *=* *12 pairs of fish in all experiments

Parameter	Calculation method	Fasted	Fed	*F*	*df*	*p* Value
State 3	Mass‐specific (pmol O_2_ s^−1^ mg^−1^ liver)	25.36 ± 0.93	18.81 ± 1.02	22.52	1,22	**<0.001**
	COX‐normalized (pmol O_2_ s^−1^ mg^−1^ liver)	24.11 ± 1.07	20.07 ± 1.07	5.60	1,18	**0.029**
	Total liver (pmol O_2_/s)	2373.44 ± 182.63	5819.67 ± 740.58	20.41	1,22	**<0.001**
State 4	Mass‐specific (pmol O_2_ s^−1^ mg^−1^ liver)	1.73 ± 0.05	1.88 ± 0.10	1.90	1,22	0.181
	COX‐normalized (pmol O_2_ s^−1^ mg^−1^ liver)	1.57 ± 0.07	2.04 ± 0.07	16.57	1,21	**0.001**
	Total liver (pmol O_2_/s)	160.93 ± 10.88	571.49 ± 62.58	41.76	1,22	**<0.001**
COX activity	Mass‐specific (pmol O_2_ s^−1^ mg^−1^ liver)	35.39 ± 0.49	31.62 ± 0.75	17.68	1,22	**<0.001**
	Total liver (pmol O_2_/s)	3312.24 ± 231.01	9599.44 ± 951.60	41.22	1,22	**<0.001**

**Figure 2:**
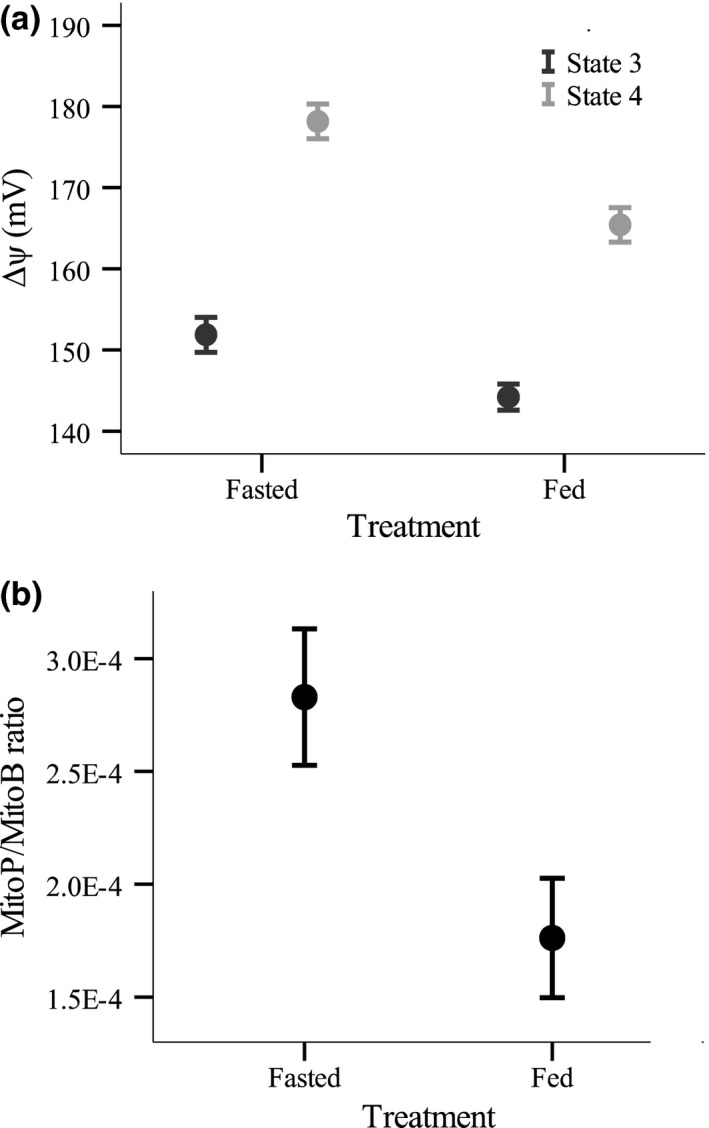
(a) Liver mitochondrial membrane potential (Δψ) and (b) hydrogen peroxide levels in vivo (estimated as the ratio of MitoP to MitoB) of brown trout (*Salmo trutta*) deprived of food or fed continuously over a 2‐week period. Values are means ± *SE*. See text for statistical analysis

The mitochondrial Δψ during both state 3 and state 4 respiration was significantly higher in the livers of fasted compared to fed fish (Figure 2a, state 3: *F*_1,20_ = 8.151, *p *=* *0.010, state 4: *F*_1,22_ = 17.786, *p *<* *0.001). In parallel with this difference, in vivo mitochondrial H_2_O_2_ levels, estimated from the MitoP/MitoB ratio, were nearly twice as high in the livers of fasted fish compared to fed controls (Figure 2b, *F*_1,19_ = 7.103, *p *=* *0.015).

## 4 DISCUSSION

We tested whether plasticity in mitochondrial metabolism in response to fasting in brown trout resulted in a reduction in the metabolic requirements of the liver and whether these changes came at a cost of increased ROS levels. We found evidence for the predicted reduction in liver metabolic requirements upon fasting, and the effect was mostly due to a dramatic reduction in liver size. When expressed in terms of oxygen consumption per unit mass of liver or normalized per unit of mitochondria, state‐specific adjustments in mitochondrial function were revealed: while the state 4 *J*O_2_ of fasted trout tended to be lower than that of fed fish, conforming to the predicted metabolic depression in response to fasting, the state 3 *J*O_2_ showed the opposite trend. These changes in mitochondrial energy demand in fasting fish were associated with both a higher mitochondrial membrane potential and higher levels of H_2_O_2_, one of the major forms of ROS. Therefore, our results provide partial support for the key hypotheses that fasting induces a reduction in metabolic requirements but at the increased risk of oxidative stress.

This study provides the first illustration of the multiple levels of biological organization that determine metabolic adjustments in response to fasting. Mitochondrial respiratory capacity has traditionally been expressed in terms of oxygen consumption per unit mass of tissue, or “number” of mitochondria (Brown & Staples, 2010; Foster & Moon, 1991; Monternier et al., 2014; Trzcionka, Withers, Klingenspor, & Jastroch, 2008). However, it is clear that when interpreting metabolic responses to fasting we must account for commonly observed reductions in organ size, notably the liver, as a consequence of glycogen depletion and/or hepatocyte autophagy (Secor & Carey, 2016; Wang et al., 2006). The mitochondrial density of the liver, as estimated by COX activity per gram of tissue, was actually higher in food‐deprived trout than in fed controls. Thus, although the liver had atrophied so that the absolute mitochondrial content of the entire liver was lower in fasted fish, this loss in the “number” of mitochondrial was partially offset by a higher mass‐specific mitochondrial density. During the metabolic transition from the fed to the fasting state, hepatic mitochondria play a crucial role that involves an increase in the oxidation of fatty acids, generating not only ATP but also ketone bodies (Rui, 2014). This suggests that the higher mitochondrial density in the livers of fasted trout may be an adaptive component of the metabolic response to food deprivation (Secor & Carey, 2016). However, our results clearly show that the reduction in the *total* “number” of hepatic mitochondria drives a decrease in liver total oxygen requirements, and presumably partly explains the reduction of whole‐body metabolism that occurs in response to food shortage (Auer, Salin, Anderson, & Metcalfe, 2016; Lamarre et al., 2016; Monternier et al., 2014).

Like many other organisms, Brown trout experience periods of limited food availability in their natural environment (Bayir et al., 2011; French, Vondracek, Ferrington, Finlay, & Dieterman, 2016; Huusko et al., 2007). The first winter is a critical period for the survival of young salmonid fishes: food is often limiting, and their survival is dependent on metabolic adjustments (Finstad, Ugedal, Forseth, & Næsje, 2004; Huusko et al., 2007). Thus, brown trout might adapt to food shortage through modification of metabolism to reduce energy demands (Auer et al., 2016). The increase in state 3 mitochondrial respiration combined with a decrease in state 4 respiration suggests a selective upregulation of the capacity to produce ATP without a concomitant increase in energy expended via proton leak (Kadenbach, 2003). The state‐specific responses of the mitochondria that we observed during fasting contrast with many studies that show comparable directional changes in state 3 and state 4 respiratory capacities (Brown & Staples, 2010, 2011; Chausse et al., 2015). Those studies were in mammals that experience much briefer periods of food limitation in comparison with the fish in this study, which may help explain the differences observed. However, a decrease in the proton leak in response to food deprivation has been reported in many species of vertebrates (Dumas et al., 2004; Rey et al., 2008; Trzcionka et al., 2008). In our study, a possible reduction in the rate of proton leakage is also supported by the higher mitochondrial membrane potential. In vivo, the respiration rates of mitochondria will never correspond exactly to the extremes of the state 3 (unlimited substrates, oxygen and ADP) and state 4 (total arrest of ATP production) situations measured in vitro, but these in vitro measurements nonetheless are likely to indicate how the mitochondria is capable of performing in the live animal. Natural and experimentally induced variation in the leakage of protons across the IMM (as measured by state 4 respiration) results in metabolic differences that have been shown to have a parallel effect on whole‐organism oxygen consumption (Salin, Villasevil, et al., 2016; Speakman et al., 2004). Decreases in the energy dissipated in offsetting the proton leak is likely to result in a reduction in the metabolic demand of the liver, which will be beneficial for the organism during periods of food shortage.

The energy‐saving benefits of the metabolic response observed in fasted fish were associated with a cost in terms of an increase in ROS levels. The livers of fasted animals had MitoP/MitoB ratios nearly twice that of fed animals, indicating higher mitochondrial H_2_O_2_ levels. This might be a direct consequence of their higher mitochondrial membrane potentials, as the rate of mitochondrial ROS production is very sensitive to the proton‐motive force (Korshunov et al., 1997; Miwa & Brand, 2003). The impact of increasing H_2_O_2_ levels in fasted trout is likely to be a shift from essential cellular signalling processes to disrupted signalling and in turn oxidative stress (Sies, 2017). However, the analysis of mitochondrial H_2_O_2_ levels by itself might be insufficient to make inferences about the levels of oxidative stress. Oxidative stress will occur only if the generation of ROS exceeds the capacities of antioxidant defence and repair mechanisms (Finkel & Holbrook, 2000; Selman et al., 2012). There may be an immediate trade‐off due to the resources required to generate antioxidant and repair systems (Monaghan et al., 2009). Alternatively, there may be a long‐term trade‐off involving increases in the rate of accumulation of oxidative damage. Previous work in brown trout has demonstrated that metabolic responses to long‐term fasting increased oxidative damage and that this higher oxidative damage levels persisted after refeeding (Bayir et al., 2011). Our findings provide an exciting opportunity for future work to test the hypothesis that high levels of ROS will drive potential long‐term costs of oxidative stress in animals responding to fluctuating food levels.

We conclude that flexibility in both liver size and mitochondrial function promotes energy‐saving during periods of food shortage in juvenile brown trout. However, this short‐term benefit is associated with a cost of higher ROS levels that could drive the established long‐term deleterious effects of periods of food shortage on animal performance (Midwood et al., 2016; Monaghan et al., 2009; Schull et al., 2016). Additionally, our results highlight the value of an integrative approach to studying metabolic responses to food shortage; measuring only energetic aspects of the mitochondria would have missed the potential long‐term costs of elevated ROS levels, while calculating mitochondrial respiration in terms of mass‐specific rates would have led to misleading interpretations of the consequences for whole‐animal metabolism. The implications of such changes in mitochondrial properties for the subsequent life history will be an important avenue for future study.

## COMPETING INTERESTS

The authors declare they have no competing interests.

## AUTHORS' CONTRIBUTIONS

K.S., C.S., C.C. and N.B.M. conceived the ideas and designed methodology. K.S., E.M.V., G.J.A., S.K.A. and W.M. collected the data. K.S., E.M.V., S.K.A. and C.C. analysed the data. K.S. led the writing of the manuscript. K.S., E.M.V., S.K.A., C.S., R.H., C.C. and N.B.M. revised the manuscript and added some comments. All authors gave final approval for publication.

## DATA ACCESSIBILITY

Data available from the Dryad Digital Repository: https://doi.org/10.5061/dryad.v0vg627 (Salin et al., 2018).
