# Supplementary material for: Decreased mitochondrial metabolic requirements in fasting animals carry an oxidative cost
Source: Funct Ecol. 2018 May 29;32(9):2149–57. doi: 10.1111/1365-2435.13125 (PMC6175143; doi:10.1111/1365-2435.13125)
Supplement: Supplementary file 1 [file FEC-32-2149-s001.docx]

**Table S1**. Parameter estimates from linear mixed models of the effects of mass-specific cytochrome *c* oxidase (COX) activity and food treatment (fasted *versus* fed) on mass-specific mitochondrial respiration rate at state 3 and state 4 of the livers of juvenile brown trout *Salmo trutta* (*N* = 12 fish per treatment group). Parameter estimates for food treatment are for fasted relative to fed fish.

| Dependent variable | Parameter | Estimate ±1SE | *d.f.* | *t* | *P* value |
| --- | --- | --- | --- | --- | --- |
| State 3 | Intercept | -2.185 ± 9.644 | 18 | -0.227 | 0.823 |
|  | COX activity | 0.664 ± 0.304 | 18 | 2.187 | 0.042 |
|  | Food treatment | 4.050 ± 1.711 | 19 | 2.367 | 0.029 |
| State 4 | Intercept | -0.796± 0.659 | 21 | -1.208 | 0.240 |
|  | COX activity | 0.085 ± 0.021 | 21 | -4.084 | 0.001 |
|  | Food treatment | -0.477 ± 0.117 | 21 | -4.071 | 0.001 |
