# Supplementary material for: Decreased mitochondrial metabolic requirements in fasting animals carry an oxidative cost
Source: Funct Ecol. 2018 May 29;32(9):2149–57. doi: 10.1111/1365-2435.13125 (PMC6175143; doi:10.1111/1365-2435.13125)
Supplement: Supplementary file 2 [file FEC-32-2149-s002.pdf]

Functional Ecology
BRITISH ECOLOGICAL SOCIETY

How metabolic adjustments in response to food scarcity can have long-term negative effects

Karine Salin, Eugenia M. Villasevil, Graeme J. Anderson, Sonya K. Auer, Colin Selman, Richard C. Hartley, William Mullen, Christos Chinopoulos and Neil B. Metcalfe

Many animals experience periods of food shortage in their natural environment. Some animals can reduce their metabolic requirements to preserve their limited resources. However, recent research suggests that, although metabolic reduction can bring quick benefits, it is also associated with a surprising variety of costs that are often not evident until much later in the animal's life. We don't understand why this short-term benefit is associated with long-term deleterious effects. One possible explanation for this compromise may lie in the mitochondria. Mitochondria are organelles found in the cells of eukaryote organisms, and are responsible for one of the most important process in life: cellular respiration. Mitochondria transform food and oxygen into energy-rich molecules of ATP (Adenosine TriPhosphate) which are used as the energy source for most biological processes. The functioning of the mitochondria is, however, a double-edged sword that on the one hand supplies energy to the cell, and on the other leads to the formation of damaging *reactive oxygen species* (ROS) that can potentially damage cells.

Our study tested whether food shortage causes: 1) the capacity of the mitochondria to supply energy to the cell to improve, so promoting energy saving, but also 2) a greater generation of ROS. We examine these mitochondrial responses in brown trout *Salmo trutta,* a fish that experiences periods of food shortage in its natural environment.

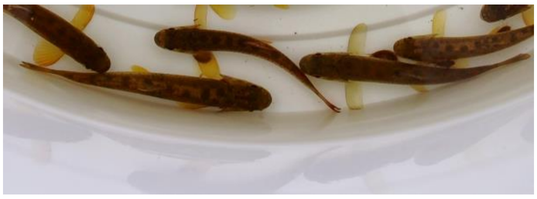

Photo provided by authors.

Our findings demonstrate that brown trout experiencing a period of food shortage show dramatic reductions in the mitochondrial requirement for oxygen, and in turn for energy substrates. This reduction in energy requirements in fasted fish, however, was associated with significantly increased ROS levels, and hence potentially a greater risk of cell damage. This work sheds light on the mechanisms by which temporary reductions in energy requirements during fasting, while providing short-term energetic benefits, can lead to a potential cost that might cause animals to experience the known long-term deleterious effects of periods of food shortage.
